# Clinical features of varicella-zoster virus caused neurological diseases detected by metagenomic next-generation sequencing

**DOI:** 10.1515/med-2023-0744

**Published:** 2023-07-10

**Authors:** Shuhua Xie, Xuying Yang, Han Xia, Jinxing Lai, Qing Liu, Zhijuan Lu, Dehai He, Xianghong Liu

**Affiliations:** Department of Neurology, The Affiliated Ganzhou Hospital of Nanchang University, Ganzhou, 341000, China; Department of Scientific Affairs, Hugobiotech Co., Ltd., Beijing, 100000, China

**Keywords:** VZV infection, central nervous system, mNGS, clinical features

## Abstract

Clinical presentation of central nervous system (CNS) infections caused by varicella-zoster virus (VZV) is highly sophisticated, making identification challenging. We retrospectively reported 18 cases of VZV neurologic disease confirmed by metagenomic next-generation sequencing (mNGS). The detection rate of mNGS was higher than that of PCR assay (100 vs 66.7%, *p* < 0.05) and serum IgM antibody (100 vs 68.8%, *p* < 0.05) measurement. Of the 18 cases, five patients were diagnosed with acute meningitis, three with acute meningitis combined with facial neuritis, three with acute meningitis combined with polycranial neuritis, and the remaining seven with various clinical diagnoses. Typical clinical symptoms included headache (15), fever (9), and rash (11). Cranial or spinal MRI showed abnormalities in 12 patients, and 17 patients had obvious neurological symptoms. The predominant genotype of VZV in this study was genotype J (100%, 10/10). All patients were treated with acyclovir/penciclovir and dexamethasone, 16 recovered and 2 died. Our study highlights the good performance of mNGS in diagnosing CNS infection caused by VZV. It could provide additional diagnostic evidence in patients with diverse clinical spectrum and variable manifestations.

## 1 Introduction

As an exclusive human alpha herpes virus, varicella zoster virus (VZV) can cause varicella (chickenpox) as the primary infection presentation, the virus can be then latent in sensory and cranial and dorsal root ganglionic neurons [1]. Spontaneously or in the presence of decreased VZV-specific cellular immunity, VZV can reactivate to produce herpes zoster (shingles) in 10–20% of the population [2,3]. The incidence of VZV reactivation in the elderly or immunosuppressed patients even reaches over 50% [4,5]. Reactivation of VZV can cause vesicular skin eruption, chronic pain (post-herpetic neuralgia), and multisystem disorders [6–8]. Severe neurological complications, inducing meningitis [9–11], encephalitis [12], meningoencephalitis [13], myelitis [14], vasculopathy [15], and other diseases [16–18], may occur when VZV spreads to the nervous system. Neurological disorders pose a fatal threat to humans, especially in elderly populations [19]. Considering that more than 90% of the population worldwide is latent with the virus [2,4], the burden of VZV neurological diseases is high.

The diagnosis of central nervous system (CNS) infection caused by VZV is usually based on the temporal association of the rash with onset, the synthesis of anti-VZV antibodies, and the presence of VZV DNA in cerebrospinal fluid (CSF) [4]. Several studies have documented that PCR assays using CSF for VZV detection showed lower sensitivity than antibody measurements [20,21]. However, the application of antibody measurements is often limited due to the narrow time windows of the presence of immunoglobulin M (IgM) antibody [22] and the uncertainty in the specificity of immunoglobulin G (IgG) antibody detection [6]. Besides, VZV infection in CNS can occur without a rash, or with a rash later in the course of a cutaneous outbreak [7,11,23–25]. A multicenter study of VZV CNS infection reported that 44% of the patients showed no skin lesions [26]. The absence of skin lesions is likely to cause misdiagnosis of VZV, inducing persistent neuropathic pain with fatal sequelae. Thus, a more sensitive and effective diagnostic method is needed.

As a target-independent method, metagenomic next-generation sequencing (mNGS) enables simultaneous and unbiased detection of almost all pathogens from clinical samples [27], which has shown high sensitivity in a variety of infections [28]. In this study, we report 18 patients with CNS infections caused by VZV. The efficacy of mNGS and conventional methods for pathogen identification has been compared. The clinical manifestations of these patients with CNS infections caused by VZV have been described.

## 2 Methods

### 2.1 Study subjects

This study retrospectively reviewed clinical data of patients with VZV neurologic diseases, who admitted to our hospital from March 2021 to July 2022. All enrolled patients met the following inclusion criteria: (1) any alteration in mental state (decreased level of consciousness, lethargy, or abnormal mental behavior for ≥24 h) or the presence of seizures; (2) at least one of the following symptoms: body temperature ≥38℃ (within 72 h before or after onset), focal neurological manifestations, CSF white blood cell count >5 × 10^6^/L, CSF cytology analysis showing lymphocytic inflammation, lesions by brain imaging indicating encephalitis, and EEG findings suggesting encephalitis; (3) positive nucleic acid for VZV (PCR or mNGS), or positive CSF and/or serum IgM antibodies. Cases diagnosed with other etiologies were excluded [29].

For each case, clinical records of prodromal symptoms, cranial or spinal imaging findings, biochemical tests, laboratory tests, and the clinical course of the disease were investigated. Additional records including treatment strategies, clinical outcomes, and relevant follow-up data were also collected.


**Ethics statement:** The studies involving human participants were reviewed and approved by the Ethical Review Committee of The Affiliated Ganzhou Hospital of Nanchang University. The participants provided their written informed consent to participate in this study. Written informed consent for minor participants was provided by either parent or guardians of participants.

### 2.2 Conventional detection

ESR-104G (VZV-IgG) and ESR-104M (VZV-IgM) (Institute Virion/Serion GmbH, Würzburg, Germany) were used for IgG and IgM antibody measurement. There exist three distinct threshold levels of serum IgM (0.470, 0.653, and 0.7583) that serve as the basis for positive judgment. A level of serum IgM below 0.470 is deemed negative, while a level between 0.470 and 0.653 is considered critical. A level between 0.653 and 0.7583 is classified as weakly positive, and a level greater than 0.7583 is deemed positive. Similarly, there are also three threshold levels of serum IgG (0.198, 0.369, and 0.469) that serve as the criteria for positive judgment. A level of serum IgG below 0.198 is classified as negative, while a level between 0.198 and 0.369 is considered critical. A level between 0.369 and 0.469 is classified as weakly positive, and a level greater than 0.469 is deemed positive.

PCR was utilized for VZV nucleic acid detection. DNA was extracted from CSF samples using DNA Extraction Mini Kit (Kaijie, Shengzhen, China). All amplifications were performed using SU PCR Mix (Nuhighbio.com, Beijing, China) and in Eppendorf Mastercycler Gradient PCR Thermal Cycler (Eppendorf, Hamburg, Germany). In the PCR reaction, 117-bp fragment of *ORF62* gene was amplified, the primers were 5-*GGAGACCAGAGAGGGTCACT*-3 and 5-*AACCCTCTAGGCCGATCTGT*-3. The PCR product was confirmed by gel electrophoresis.

### 2.3 mNGS detection

CSF samples were collected from patients and stored at −20℃. The samples were sent for mNGS detection (Hugobiotech, Beijing, China). DNA was extracted and purified following the standard procedures of QIAamp DNA Micro Kit (QIAGEN, Hilden, Germany). Qubit 4.0 (Thermo Fisher Scientific, MA, USA) was used for DNA concentration and quality control. Libraries were constructed using QIAseq Ultralow Input Library Kit (QIAGEN, Hilden, Germany). After qualified, libraries were sequenced on Nextseq 550 platform (Illumina, San Diego, USA).

### 2.4 Bioinformatics and statistical analysis

The generated raw reads after mNGS sequencing were filtered by removing adapters, low-quality and short reads (<35 bp), and the clean data were obtained. Bowtie2 was used for excluding human sequences by mapping the clean data to the human reference genome (hg38). For microorganism identification, the remaining reads were then aligned to the published microbial genome databases, which were downloaded from National Center for Biotechnology Information (fttp://ftp.ncbi.nlm.nih.gov/genomes/). Genotype analysis was performed based on metagenomic data. For VZV, if the number of VZV-specific sequence is greater than or equal to three and not detected in the no template control, it will be judged as mNGS positive.

Frequencies, median, and summary data of patients’ clinical characteristics were calculated by IBM SPSS 25.0. The count data were expressed in case or percentage, *t*-test or chi-square test was used in the comparisons between the two groups, *p*  <  0.05 was taken as statistically significant.

## 3 Results

### 3.1 General information and clinical characteristics

This study focused on 18 participants with VZV neurologic diseases, including ten males (55.6%) and eight females (44.4%). The ages of these patients ranged from 15 to 85 years old, with a median age of 57 years old. Of the 18 patients in our study, five patients were diagnosed with acute meningitis, three with acute meningitis combined with facial neuritis, three with acute meningitis combined with polycranial neuritis, one with acute meningitis combined with myelitis, choriomeningitis, and myeloradiculitis, one with acute meningoencephalitis, one with acute meningoencephalitis combined with bilateral facial neuritis, one with acute meningoencephalitis combined with polycranial neuritis, one with acute meningoencephalitis combined with conjunctivitis and blepharitis, one with acute brainstem encephalitis complicated with myelitis, and one with acute choriomeningitis and myelitis. Typical clinical symptoms included headache (15), fever (9), and rash (11), another two patients developed fever after pulmonary or other organ infection. In addition, ten patients had underlying diseases including hypertension (3), diabetes (3), autoimmunity disease (2), cerebrovascular disease (1), and beta-thalassemia (1). The CSF leukocyte counts were remarkably elevated in 13 patients, revealing lymphocyte predominance, and the CSF protein levels increased in 13 patients. Twelve patients had abnormal brain or spinal cord MRI, and 17 patients had obvious neurological symptoms. The clinical characteristics of all subjects are listed in Table 1.

**Table 1:** Clinical characteristics of all subjects

Patient/sex/age (years)	CSF indicators		Abnormal MRI	Skin rash	Clinical characteristics	Neurological signs and symptoms	Underlying disease	Onset to admission (days)	Clinical diagnosis
	WBC (cells/mm^3^)	Pro (mg/dL)							
1/F/67	6	95.19	+	+	Body pain, bilateral lower extremity weakness	Nuchal rigidity	—	25	Acute meningitis, myelitis, spinal meningitis, myeloradiculitis
2/M/19	132	35.94	+	+	Headache, dizziness, vomiting	Right occipital and right post-auricular pain, incomplete eyelid closure, tongue numbness	—	10	Acute meningitis, facial neuritis
3/F/63	34	32.57	+	−	Severe pain in the right head and face	Pharynx reflex was weakened, high pain at the right temporal lobe	Cerebral artery infarction	7	Acute meningitis, polycranial neuritis
4/F/49	269	114.01	+	−	Headache, fever, fatigue on both lower limbs	Nuchal rigidity, positive Kernig and Babinskis sign	Diabetes	12	Acute choriomeningitis, myelitis
5/M/49	327	199.61	−	+	Headache	Nuchal rigidity	—	3	Acute meningitis
6/F/59	366	183.60	+	−	Headache, fever	Nuchal rigidity, blunted response	—	8	Acute meningitis
7/F/62	189	297.00	+	+	Headache, body pain, fever, vomiting	Nuchal rigidity	Autoimmunity disease	7	Acute meningitis
8/M/15	459	76.10	+	+	Headache, fever, vomiting	Nuchal rigidity	Beta-thalassemia	3	Acute meningitis
9/M/62	593	144.7	+	−	Headache, vomiting, limb twitch	Suspect positive Kernig sign	—	4	Acute meningoencephalitis, bilateral facial neuritis
10/F/32	257	77.63	+	−	Headache, fever, cough	Right mouth angle deviation, left nasolabial sulcus was shallow	—	14	Acute meningitis, facial neuritis
11/F/55	100	43.63	+	−	Headache, pharyngalgia, otalgia, hoarseness with dysphagia	Dysarthria and bucking, positive Kernig sign, nuchal rigidity	Diabetes	4	Acute meningitis, polycranial neuritis
12/M/85	140	166.52	+	+	Headache, dizziness, right-sided tinnitus	Nuchal rigidity, right eye fissure became larger, right mouth angle drop, hearing loss in right ear	Hypertension, obsolete tuberculosis, rectal cancer, intracerebral hemorrhage	7	Acute meningoencephalitis, polycranial neuritis
13/M/46	357	319.88	+	−	Headache	Right temporal pain	Hypertension	3	Acute meningoencephalitis
14/M/47	6	311.4	+	+	Neck pain, four limbs fatigue, cardiac arrest, rashes all over body	Lost autonomous respiration, quadraparesis	—	10	Acute brainstem encephalitis, myelitis
15/F/77	28	58.54	−	+	Inability to close the left eyelid	Crooked angulus oris, left nasolabial sulcus was shallow	Diabetes	8	Acute meningitis
16/M/76	0	43.67	−	+	Pain in the right head and face	Difficulty in opening right eyes	—	12	VZV meningoencephalitis, conjunctivitis, blepharitis
17/M/47	6	55.96	−	+	Headache	No apparently positive signs	Autoimmunity disease	1	Acute meningitis
18/M/71	5	33.01	−	+	Headache, dizziness, pain in right ear	Traction pain in the right auricle, right auricle swelling	Hypertension	4	Acute meningitis, facial neuritis

### 3.2 Diagnostic performance of mNGS and treatment strategies

mNGS and PCR were performed on all 18 CSF samples, while serum VZV IgG and IgM antibodies were examined in 16 patients. According to the mNGS detection, VZV unique reads were detected in all cases with specific sequences ranging from 3 to 259,789, the relative abundance ratio of VZV ranged from 0.03 to 99.99%, and its coverage ranged from 0.18 to 99.95%. Ten cases were with more than 90% VZV coverage by mNGS detection. Furthermore, genotype J was identified in all these ten cases, indicating genotype J as the major genotype of VZV in this study.

VZV IgG antibodies were present in serum samples from 15 (15/16, 93.8%) patients, and VZV IgM antibodies were observed in 11 (11/16, 68.8%) patients. Twelve (12/18, 66.7%) patients had positive PCR results for VZV in CSF. The detection rate of mNGS for VZV was higher than that of PCR assay (100 vs 66.7%, *p* < 0.05) and serum IgM antibody (100 vs 68.8%, *p* < 0.05) measurement. All patients were treated with acyclovir/penciclovir and dexamethasone. Two cases died, the others were discharged and fully recovered after 1-month follow-up. The yield of different etiological examinations, therapy, and prognosis of all patients are summarized in Table 2.

**Table 2:** Yield of different etiological examinations, therapy, and prognosis of VZV-infected CNS

Patient	Conventional detection			mNGS	Specific sequences	Relative abundance (%)	Coverage (%)	Genotype	Treatment (days)	Hospital days	Condition at discharge	Follow-up (after discharge 1 month)
	Serum IgM (U/mL)	Serum IgG (U/mL)	CSF PCR									
1	− (0.044)	+ (1.797)	−	+	30	0.34	1.4	—	ACV (11), PD (10), immune globulin	28	Symptomatic relief	Recovered
2	+ (0.862)	+ (1.893)	−	+	15	0.58	0.75	—	ACV (15), DEX (3)	24	Symptomatic relief	Recovered
3	− (0.171)	+ (1.747)	+	+	2,112	34.01	68.49	—	ACV (7), DEX (3)	12	Symptomatic relief	Recovered
4	+ (0.928)	+ (2.571)	+	+	145	2.38	7.30	—	ACV (15), DEX (3)	29	Symptomatic relief	Recovered
5	+ (1.017)	+ (2.556)	+	+	131,720	99.99	99.54	J	ACV (21), DEX (5)	16	Symptomatic relief	Recovered
6	+ (0.813)	+ (1.716)	+	+	83,660	99.95	97.31	J	ACV (16), DEX (5)	2	Symptomatic relief	Recovered
7	+ (0.951)	+ (1.246)	+	+	259,789	99.98	99.97	J	ACV (20), DEX (5)	23	Asymptomatic	Recovered
8	+ (1.113)	+ (2.844)	+	+	61,683	99.51	99.91	J	ACV (19), DEX (5)	23	Asymptomatic	Recovered
9	+ (0.894)	+ (1.681)	+	+	41,451	99.02	99.94	J	ACV (16), DEX (5)	21	Asymptomatic	Recovered
10	+ (0.997)	+ (1.797)	+	+	9,756	86.18	98.46	J	ACV (21), DEX (5)	22	Symptomatic relief	Recovered
11	+ (1.213)	+ (2.912)	−	+	3	0.03	0.18	—	ACV (15), DEX (3)	15	Symptomatic relief	Recovered
12	− (0.213)	+ (1.516)	+	+	73,220	98.59	99.94	J	ACV (29), DEX (5)	32	Symptomatic relief	Recovered
13	− (0.337)	− (0.019)	+	+	80,802	96.45	99.95	J	ACV (20), DEX (5)	22	Asymptomatic	Recovered
14	+ (0.854)	+ (1.986)	+	+	12,527	97.21	99.78	J	ACV (6), DEX (5)	7	Respiratory failure	Died
15	+ (1.010)	+ (2.247)	−	+	20	0.26	0.93	—	ACV (15), DEX (5)	19	Septic shock, cerebral infarction, multiple organ dysfunction	Died
16	− (0.229)	+ (2.973)	+	+	21,254	83.37	99.85	J	ACV (4), DEX (5)	6	Symptomatic relief	Recovered
17	N	N	−	+	2,005	28.08	54.44	—	ACV (6), DEX (6)	8	Symptomatic relief	Recovered
18	N	N	−	+	19	7.67	1.06	—	PCV (15), DEX (8)	14	Asymptomatic	Recovered

“+” stands for positive; “−” stands for negative; “N” represents no data; “ACV” stands for acyclovir; “PD” stands for prednisone; “Ig” stands for immune globulin; “PCV” stands for penciclovir; “DEX” stands for dexamethason.

## 4 Case descriptions

**Figure 1:**
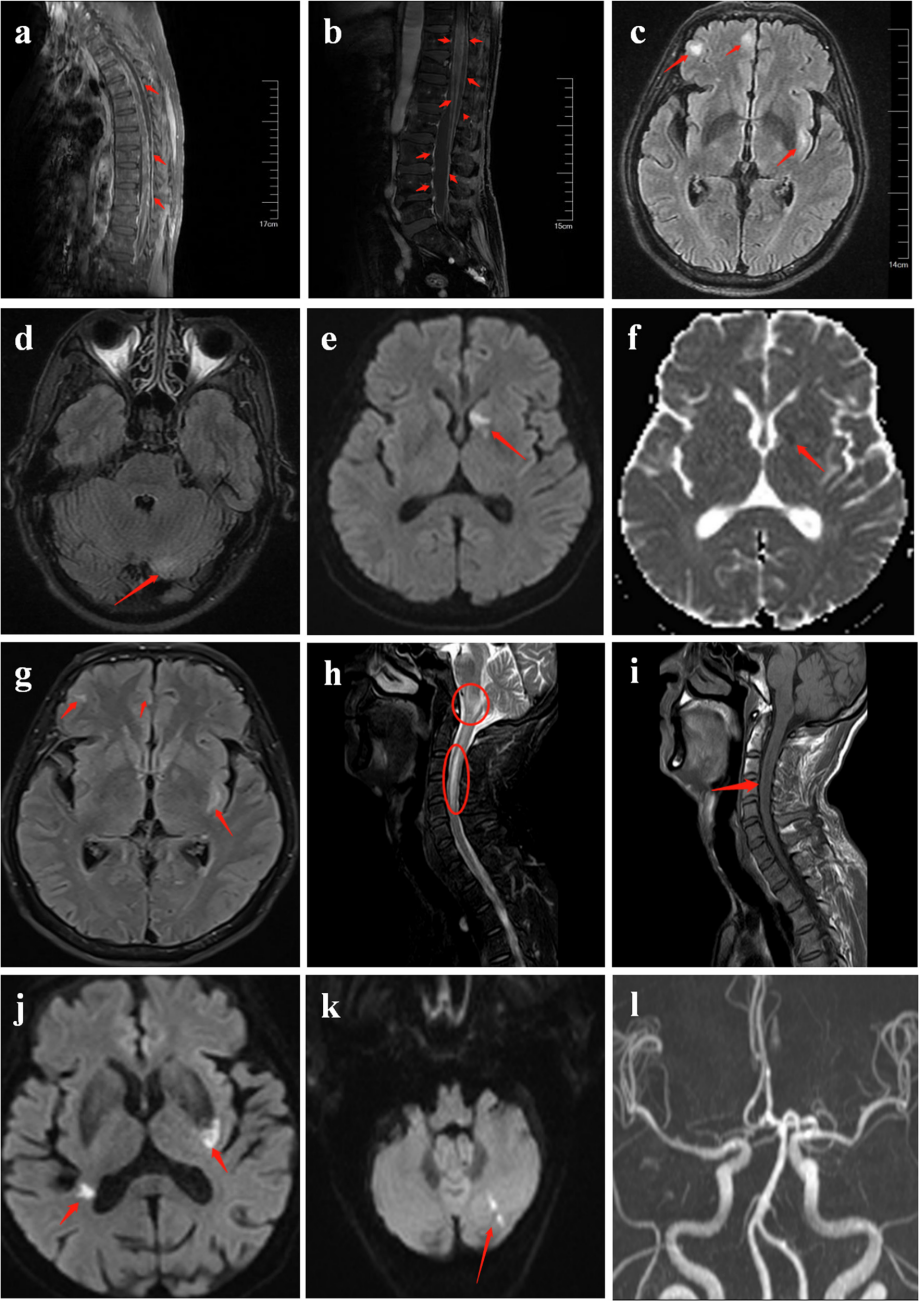
Neuroimaging data of the patients. (a and b) Thoracic and lumbar spine MRI of case 1 showing diffuse irregular thickening of the myelomeningocele. (c and d) Brain MRI of case 9 showing bilateral frontotemporal, parietal, left insula, and multiple nodular in the bilateral cerebellum hemispheres. (e–g) Brain MRI of case 9 showing new patchy foci in the left basal ganglia were appreciated or acute infarction. (h and i) Brain and cranial MRI of case 14 showing abnormal signal of the pontine, medulla oblongata, and cervical spinal cord at the C2–C5 levels. (j–l) Brain MRI of case 15 revealing acute infarction of cerebellar hemispheres, frontal lobe, basal ganglia, corona radiata, the right basal ganglia, and multiple spot shadows in the lateral ventricles.

Case 1, a female patient aged 67, was admitted to the hospital on December 27, 2021, with generalized body ache and marked weakness in both lower extremities for 10 days. She developed pain in both lower limbs without obvious inducement on December 7, 2021, mainly in below knee with upper back dull pain and weakness in both of her lower limbs. She was treated at a native Hospital on December 12, 2021. On the next day, she developed herpes in the sacrococcygeal region, with significant pain in the lower back and progressive radiating pain in the bilateral lower extremities. She was transferred to the emergency department of our hospital because of the gradual worsening of her condition (presented with dysuria and constipation gradually). The patient was preliminary diagnosed with acute myelitis. Routine blood examination reported that percentage of neutrophils (82.20%) and C-reactive protein (4.50 mg/L) were elevated. A lumbar puncture was done on December 29, 2021; the results showed an elevation in protein (95.19 mg/dL) and a normal CSF pressure (175 mmH_2_O). Thoracic and lumbar spine MRI enhanced scans are shown in Figure 1a and b, which showed diffuse irregular thickening of the myelomeningocele. On January 8, 2022, the patient was transferred to the department of neurology, mNGS was performed, suggesting VZV infection. The patient was given anti-viral, anti-inflammatory, pain relief, and neurotrophic therapy, her prior symptoms (pain and numbness and weakness in both of his lower limbs) improved, and she was finally discharged.

Case 9, a male patient aged 62, was admitted to the hospital on March 6, 2021 with headache and vomiting for 4 days and limb twitches for 3 days. He had a history of headache, which increased in severity and extent during this episode. The patient visited a local hospital with a headache of no apparent trigger on March 2, 2021. During the process of hospitalization, muscle twitches of the right upper extremity were observed, followed by twitching of the limbs, unconsciousness, trismus, and upturn in both eyes, these symptoms relieved after a duration of approximately 3 min. He was transferred to our hospital due to aggravation of the conditions. Biochemical and cytological examination of CSF showed an elevated white blood cell count (593 cells/mm^3^) and a total protein of 144.7 mg/dL. Brain MRI showed multiple nodules in the bilateral frontotemporal and parietal lobes, left insula, and bilateral cerebellum hemispheres (Figure 1c and d). Viral encephalitis was suspected. Acyclovir and antiepileptic agents were given to this patient. On March 13, 2021, CSF mNGS reported 41,451 specific reads mapping to VZV. Dexamethasone was administered, and his headache improved significantly. On March 13, 2021, the patient developed incomplete eyelid closure and a shallow nasolabial fold, VZV invading the geniculate ganglion of bilateral facial nerves was considered. Re-examination of brain MRI revealed a new plaque-like lesion or acute infarct in the left basal ganglia on March 21, 2021 (Figure 1e–g), which was judged to be a vasculitis infarct caused by VZV infection. After anti-viral, anti-inflammatory, antiepileptic, and other symptomatic supportive treatments, the patient recovered and was discharged.

Case 14, a male patient aged 47, was admitted to the hospital on April 14, 2022, with neck pain for 10 days and limb weakness for 2 days. The patient had no specific medical history. On April 4, 2022, he woke up with neck pain without any obvious inducements and then developed a scattered rash all over his body the next day. On April 12, 2022, the patient presented to a local hospital with weakness in his extremities, which was later considered as myelitis (Figure 1h and i). He had a sudden loss of consciousness, respiratory arrest, and slowed heartbeat upon return to the unit. After emergency endotracheal intubation with mechanical ventilation, cardiopulmonary resuscitation, and symptomatic treatment, he resumed autonomous cardiac but lost autonomous respiration. He was then transferred to our hospital. Routine blood tests showed a neutrophil percentage of 87.5%, a lymphocyte percentage of 8.5%, an albumin of 39.28 g/L, a d-dimer of 1.54 mg/L, and a procalcitonin level of 0.83 ng/mL. CSF analyses demonstrated elevated protein (311.4 mg/dL) and glucose level (6.39 mmol/L). He was diagnosed with brainstem encephalitis and myelitis, and was treated with intravenous injection of acyclovir and dexamethasone. On April 15, 2022, the patient developed fever, sputum production, and rales on auscultation of the lungs. Aspiration pneumonia was considered, and the patient was given piperacillin/tazobactam (4.5 g, q8h). On April 17, 2022, CSF mNGS revealed 12527 VZV specific reads. ECG showed sinus bradycardia, and a temporary pacemaker was inserted to assist the rhythm. Despite aggressive resuscitation, the patient failed to breathe by himself, his family abandoned treatment on April 22, 2022.

Case 15, a female patient aged 77, was admitted on April 4, 2022, with recurrent dizziness for half a month, aggravated left eyelid opacification, and orofacial distortion for 8 days. She suffered from dizziness and nonprojectile vomiting after catching a cold on March 20, 2022. She was hospitalized in our emergency department and was diagnosed with benign paroxysmal positional vertigo. Her symptoms improved and was discharged on March 27, 2022. After discharge, she still presented with incomplete closure of the left eye and mouth angle askew to the right side, and the discomfort and dizziness continued. The patient was readmitted after self-administered proprietary Chinese medicine, which had no significant effect. The patient was diagnosed with Ramsey-Hunt syndrome. She was treated with pitavastatin and promethazine for dizziness control, dexamethasone sodium phosphate for anti-inflammatory, acyclovir for anti-viral treatment, vitamin B1, and mecobalamin for neuronutrition, but she still felt dizziness. On April 5, 2022, the patient experienced dysphagia and was unable to ingest much food, but her family rejected nasogastric tube feeding. On April 12, 2022, the patient presented with neck stiffness and positive meningeal irritation signs. On April 13, 2022, CSF analyses demonstrated elevated protein levels (58.54 mg/dL) and an elevated glucose level (6.39 mmol/L). On April 14, 2022, the patient had loss of consciousness, cold extremities, low blood pressure, increased heart rate, tachypnoea, and shock. The PCT, routine hemogram, coagulation factor level, serum electrolytes, kidney and liver function of this patient were also abnormal. Cardiac color Doppler ultrasonography showed an atrial septal defect. The patient was diagnosed with septic shock and atrial septal defect, biapenem combined with linezolid was added for antiviral treatment. On April 15, 2022, the patient presented with right-sided extremity weakness, her brain MRI revealed acute infarction of cerebellar hemispheres, frontal lobe, basal ganglia, corona radiata, the right basal ganglia, and multiple spot shadows in the lateral ventricles (Figure 1j–l). In the meanwhile, mNGS analysis revealed a low load of VZV in the CSF. Subsequently, the patient was treated with acid correction, hypervolemia, rehydration, anti-infection, improving circulation, anti-viral agents, and electrolyte replenishment therapy, but she eventually died on April 23, 2022 due to multi-organ failure.

## 5 Discussion

The crucial diagnostic sign of VZV CNS infections is the presence of specific viral DNA in the CSF or blood. In this study, we described 18 patients with CNS infection caused by VZV. The advantages of mNGS over traditional microbiological assays for diagnosis of VZV were highlighted, consistent with a previous study [21]. Most of the enrolled cases had CSF cytology, suggesting a higher likelihood of viral infection. Clinical symptoms and radiographic features of viral infection can also be observed in some patients. Hence, there may be some selection bias of cases, which may also contribute to the high detection rate of mNGS in our study. The anti-VZV IgG antibody test in CSF is reported to be even more sensitive in diagnosing VZV vasculopathy than PCR [20]. However, this tendency was not demonstrated in the present study due to the small number of cases. mNGS can not only help pathogen identification, but also allows homology analysis and genotyping [30]. By analyzing the SNPs of VZV, genotype J was found to be the main VZV genotype in this study. This finding is consistent with the study of Liu [31], who noted that all 19 Chinese VZV strains could be grouped into genotype J. These results may have some implications for the prevention and control of VZV.

Varicella is usually the primary infection symptom of VZV, and VZV is latent in all neuraxis-wide ganglionic neurons [32]. VZV is responsible for both varicella and herpes zoster, and is the most common cause of encephalitis and viral meningitis, surpassing herpes simplex virus [33,34]. It has become evident that VZV may affect anywhere in the neuraxis, and this disease includes a wide spectrum of different CNS manifestations [35]. In case 1, myelitis and choriomeningitis was the first manifestation, followed by lumbosacral pain. The patient’s condition progressively worsened, and the pathogen invaded deep into the brain parenchyma, causing widespread meningoencephalitis. The patient exhibited various clinical presentations caused by VZV infection. Alternatively, even diagnosed with the same disease, the patients might experience various MRI imaging manifestations and neurological signs. In this study, 8 of 12 acute meningitis patients demonstrated abnormal enhancement of the meninges, and four patients showed no abnormalities on MRI of the brain. Among the patients with acute meningoencephalitis, brain MRI revealed abnormalities in the parenchyma and the meninges in two cases: one with mild enhancement and one with no abnormalities. A complex and varied imaging presentation of VZV infection was demonstrated in this study. Therefore, the importance of early diagnosis and treatment cannot be overstated.

As a typical clinical feature of VZV infection, the rash may be absent in many cases. The absence of rash increases the difficulty of diagnosis. In this study, there were seven (38.9%) VZV patients who exhibited no rash. For instance, patient 9 presented with headache, without fever or rash as initial symptoms. The initial diagnosis of the patient was meningoencephalitis, but he developed bilateral cranial neuritis and then secondary to cerebral vasculitis. He had an elaborate spectrum of clinical presentations and complex diagnosis. Due to the diversity of clinical manifestations, the clinical diagnosis of VZV can be difficult, especially in the absence of varicella. The introduction of mNGS confirmed VZV infection in this case. This case indicated that bilateral cranial neuritis caused by VZV can be an early clinical symptom, as well as a complication of recovery period. Comparing with PCR and IgM test, mNGS revealed significant advantages in diagnosing VZV infections, especially in the absence of a rash. For example, one patient who developed a rare asymptomatic infection-induced paraplegia after anti-retroviral therapy, was confirmed as VZV-induced thoracic myelomyelitis by using CSF mNGS [36]. Fang et al. have reported a 28-year-old HIV-infected patient who presented with fulminant CNS infection without any typical VZV related rash, VZV infection was unexpectedly identified by mNGS [37]. Currently, mNGS is not widely adopted as a primary clinical diagnostic tool due to its high cost. Nevertheless, we contend that mNGS should be given priority in cases where patients exhibit severe or atypical symptoms. This study shows that 7 of the 18 patients did not exhibit rash or symptoms suggesting VZV infection. Considering clinical heterogeneity, especially in the absence of cutaneous lesions, we should pay close attention to the various symptoms associated with VZV infection.

Drugs approved for the treatment of VZV-associated disease include acyclovir, its oral prodrug valacyclovir, and famciclovir, a penciclovir prodrug [38]. Short course corticosteroids are also recommended to the antiviral therapy and may improve the outcomes [4,39]. Vaccination against VZV is currently recommended to reduce the burden of herpes zoster and post-herpetic neuralgia [40]. The patients described in this study were all middle- and old-aged, so it is difficult to elicit the history of prior vaccination. Except case 17, the remaining patients received empirical antiviral therapy for 1–10 days. Upon definitive diagnosis of VZV infection, all patients received aggressive and definitive antiviral and hormonal therapy. Unfortunately, two patients (patient 14 and 15) died due to secondary infections and organ failures. Patient 14 was severely diseased due to delay of optimal treatment for 1 week since onset of symptoms, which ultimately led to the death of the patient. The older age, severe infections, and multiple complications were the main causes of mortality in case 15. These cases demonstrate the high risk of VZV infection and emphasize the significance of early diagnosis and accurate therapies.

This study also has some limitations. This is a single-center retrospective study, and some clinical data in our cases were incomplete, which may reduce its clinical significance. The number of cases in this study is relatively small and there is a lack of controls for healthy individuals or cases of CNS infection with other pathogens. Further prospective research with a larger sample size is warranted.

## 6 Conclusions

In the article, we represent 18 cases of VZV CNS infection, which demonstrate different clinical manifestations and various imaging characteristics. The diagnosis in some cases is difficult and may lead to death if delayed. mNGS allows an effective and rapid diagnosis for viral infection.
